# Kaempferol inhibits hepatitis B virus replication *via* ERK/FOXO1 pathway-mediated suppression of the viral core promoter

**DOI:** 10.3389/fcimb.2026.1780484

**Published:** 2026-02-19

**Authors:** Wanyu Deng, Zhen Luo, Haifei Yu, Fu Chen, Jiefeng Ding, Chun Dai, Xiaoyong Zhang, Bo Qin, Jie Dou, Min Guo

**Affiliations:** 1 College of Life Science, Shangrao Normal University, Shangrao, China; 2 Department of Tibetan Medicine, University of Tibetan Medicine, Lhasa, China; 3 State Key Laboratory of Natural Medicines, School of Life Science & Technology, China Pharmaceutical University, Nanjing, China; 4 Shaoxing Women and Children’s Hospital, Shaoxing, China; 5 Department of General Surgery, Yangzhong People’s Hospital Affiliated to Medical College of Yangzhou University, Yangzhong, China; 6 Department of Infectious Diseases, Nanfang Hospital, Southern Medical University, Guangzhou, China; 7 State Key Laboratory of Organ Failure Research, Key Laboratory of Infectious Diseases Research in South China, Ministry of Education, Guangdong Provincial Key Laboratory for Prevention and Control of Major Liver Diseases, Guangdong Provincial Clinical Research Center for Viral Hepatitis, Guangdong Institute of Hepatology, Guangdong Provincial Research Center for Liver Fibrosis Engineering and Technology, Guangzhou, China

**Keywords:** core promoter, ERK, FOXO1, hepatitis B virus, kaempferol, phosphorylation

## Abstract

**Introduction:**

Chronic hepatitis B virus (HBV) infection continues to pose a significant global health burden, and current therapies rarely target the viral covalently closed circular DNA reservoir. Kaempferol (KP), a major flavonoid found in various herbs and plants, exhibits diverse bioactivities, but its potential anti-HBV activity remains unclear. This study aims to investigate the anti-HBV potential of KP and to elucidate its underlying mechanisms.

**Methods:**

The HBV-infected Huh7D^hNTCP^ cell, viral stable transfection cell HepG2.2.15, as well as a hydrodynamic injection-based chronic HBV infection mouse model, were established to evaluate the antiviral effects of KP. The levels of HBV RNAs, DNA and proteins were detected using ELISA, western blot, qPCR, immunofluorescence and immunohistochemistry. To investigate the mechanisms, viral promoter activities were assessed *via* dual-luciferase reporter assays, and relevant transcription factors were validated through qPCR and western blot analysis.

**Results:**

KP dose- and time-dependently reduced the levels of viral antigens, RNA, and DNA *in vitro*, and also significantly lowered viral markers and attenuated HBV-induced hepatic pro-inflammatory cytokines expression *in vivo*. Furthermore, KP acted in combination with the nucleoside analog entecavir to suppress HBV replication. Mechanistically, KP strongly inhibited the transcriptional activity of the HBV core promoter (Cp), and enhanced the phosphorylation of both extracellular signal-regulated kinase (ERK) and its downstream target forkhead box protein O1 (FOXO1). Importantly, the ERK-specific inhibitor U0126 completely abolished the antiviral effects of KP, confirming that its antiviral activity depended on the ERK/FOXO1 pathway.

**Discussion:**

Collectively, our results indicate that KP activates ERK-dependent FOXO1 phosphorylation, leading to transcriptional repression of the HBV Cp and thereby suppression of viral replication. These findings identify KP as a potential candidate for developing novel therapeutics against chronic HBV infection.

## 1 Introduction

Hepatitis B virus (HBV) infection remains a major global health burden, with an estimated 254 million individuals living with chronic infection worldwide. It is responsible for approximately 1.5 million new infections and 820,000 deaths annually, primarily due to complications such as liver cirrhosis and hepatocellular carcinoma (HCC) (Burki, 2024). Current first-line therapies, nucleos(t)ide analogs and pegylated interferon-alpha, which effectively suppress viral replication by targeting the viral polymerase (Pol) or modulating host immune responses, thereby reducing the risks of cirrhosis and HCC. However, these regimens rarely achieve a functional cure, defined as sustained loss of hepatitis B surface antigen (HBsAg), largely because they fail to eliminate or permanently silence the viral covalently closed circular DNA (cccDNA) (Jeng et al., 2023; Moini and Fung, 2022). This episomal minichromosome serves as a persistent transcriptional reservoir in the nuclei of infected hepatocytes and is central to viral persistence and relapse (Jeng et al., 2023; Nassal, 2015). Consequently, novel agents capable of directly targeting viral cccDNA or suppressing its transcriptional activity are urgently needed to advance curative strategies.

Following entry into hepatocytes *via* the sodium taurocholate cotransporting polypeptide (NTCP) receptor, HBV relaxed circular DNA is repaired in the nucleus to form cccDNA. This stable template drives the transcription of all viral RNAs, including pregenomic RNA (pgRNA) and subgenomic mRNAs (Tsukuda and Watashi, 2020), under the control of viral and host proteins (Kar et al., 2023; Van Damme et al., 2021). A key regulatory element for cccDNA transcription is the HBV core promoter (Cp), which governs the expression of both pgRNA and precore RNA (preC RNA). The pgRNA serves as the template for reverse transcription and encodes the hepatitis B core antigen (HBcAg) and viral viral Pol, while preC RNA is translated into the hepatitis B e antigen (HBeAg) (Quarleri, 2014). Notably, Cp mutations or deletions are clinically associated with enhanced viral pathogenicity and aggravated liver disease (Kumar, 2022). The activity of Cp is finely modulated by various host transcription factors. For instance, inhibitor of differentiation/DNA-binding 1 suppresses HBV transcription by forming a heterodimer with E2F4 and interfering with E2F4-mediated Cp activation (Wei et al., 2022). Similarly, Maf bZIP transcription factor F binds directly to Cp, competitively displaces the host factor HNF-4α from cccDNA, and reduces pgRNA synthesis (Ibrahim et al., 2021). Another repressor, SRY-related high mobility group-box 9, inhibits Cp activity *via* its high-mobility-group domain, thereby suppressing HBV replication both *in vitro* and *in vivo* (Yang et al., 2020). These observations collectively underscore the therapeutic promise of targeting the Cp-host factor interface to disrupt viral replication.

**Figure 1:**
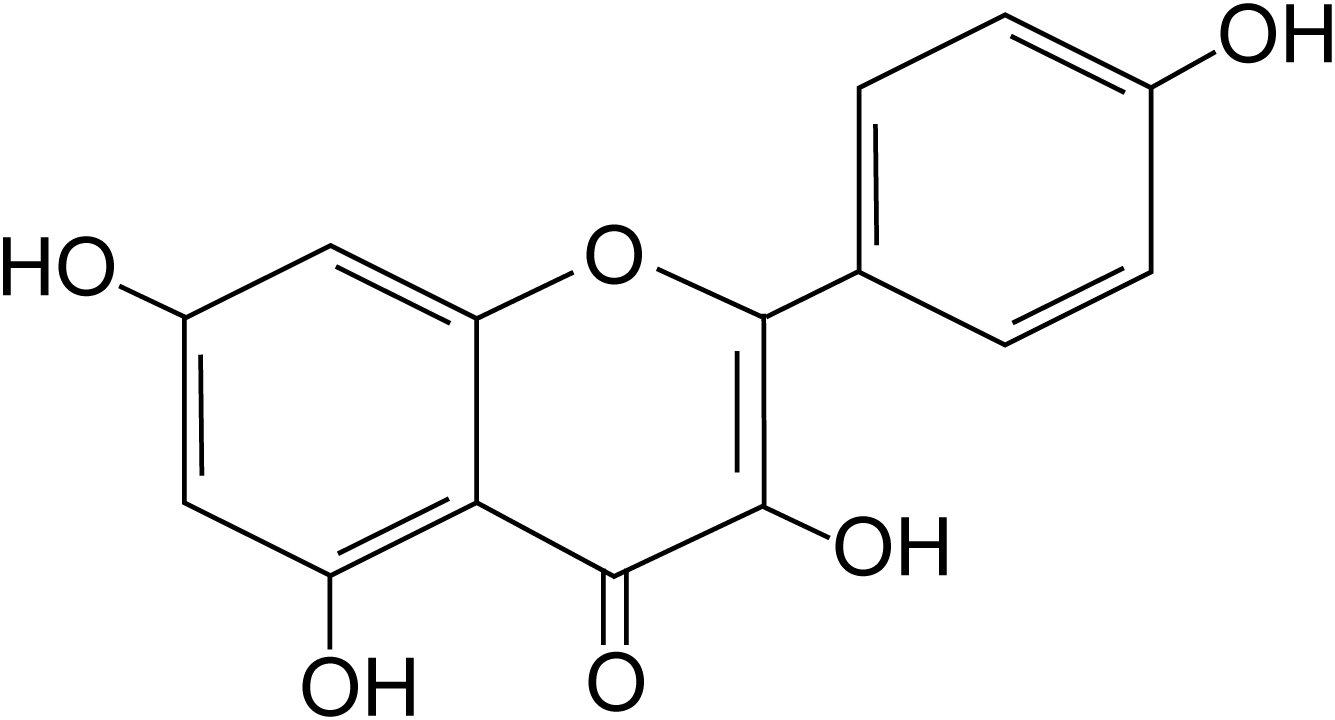
Molecular structure of kaempferol (KP).

Kaempferol (KP, 3,5,7-trihydroxy-2-(4-hydroxyphenyl)-4H-chromen-4-one, **Figure 1**) is a natural flavonoid abundantly present in various dietary and medicinal plants (Periferakis et al., 2022). It exhibits a wide range of bioactive properties, including hepatoprotective effects mediated through the reduction of hepatic lipid accumulation, attenuation of inflammation and oxidative stress, downregulation of fibrogenic pathways, and modulation of gut microbiota (Nizinski et al., 2025; Bangar et al., 2023). KP also possesses broad-spectrum antiviral activity by interfering with multiple steps of the viral life cycle, such as viral attachment/entry, transcription, and polymerase function (Periferakis et al., 2023). For example, KP inhibits Epstein-Barr virus replication by repressing the activity of viral immediate-early gene promoters (Wu et al., 2022). Against severe acute respiratory syndrome coronavirus 2, KP disrupts viral fusion by targeting the heptad repeat domains and suppresses the activity of the viral RNA-dependent RNA polymerase (Gao et al., 2023; Medoro et al., 2024). Despite these documented antiviral properties, the potential effect of KP against HBV and its underlying mechanism remain completely unexplored.

In this study, we investigate the anti-HBV activity of KP and elucidate its molecular mechanism. Using both the HBV-transfected HepG2.2.15 cell line and the HBV-infected Huh7D^hNTCP^ cell model, we demonstrate that KP potently suppresses cccDNA-driven viral transcription and replication. Mechanistically, we find that KP promotes the phosphorylation of extracellular signal-regulated kinase (ERK), which in turn induces the phosphorylation and subsequent downregulation of the transcription factor forkhead box protein O1 (FOXO1). This reduction in FOXO1 level leads to the inhibition of HBV Cp activity. The critical role of the ERK/FOXO1 axis is confirmed by the finding that the ERK-specific inhibitor U0126 completely abrogates the antiviral effects of KP. Furthermore, in a hydrodynamic injection-based mouse model of persistent HBV replication, oral gavage of KP significantly reduces serum and hepatic levels of HBV DNA, total RNA, pgRNA, and viral antigens. Our findings not only delineate a novel mechanism by which KP modulates the ERK/FOXO1/Cp axis to suppress HBV, but also highlight KP as a promising candidate for the development of novel therapeutics aimed at achieving a functional cure for chronic hepatitis B.

## 2 Materials and methods

### 2.1 Cell culture and reagents

The human hepatoma cell lines HepG2.2.15 (stably transfected with HBV), Huh7, and Huh7D^hNTCP^ (stably expressing the HBV receptor NTCP) were kindly provided by the Wuhan Institute of Virology, Chinese Academy of Sciences. All cells were maintained in Dulbecco’s Modified Eagle’s Medium supplemented with 10% fetal bovine serum at 37 °C under 5% CO_2_. KP and Entecavir (ETV) were purchased from Chengdu Alfa Biotechnology and Merck Life Science, respectively. Both compounds were dissolved in dimethyl sulfoxide as stock solutions, with the final dimethyl sulfoxide concentration in all treatments kept below 0.1%. The ERK inhibitor U0126 was obtained from MedChemExpress. The HBV promoter luciferase reporter plasmids, including pGL3-Cp, pGL3-Xp, pGL3-SpI, pGL3-SpII, were kindly provided by Prof. Mengji Lu from Institute of Virology, University Hospital of Essen, Germany and were constructed as previously described (Zhang et al., 2011). The pAAV-HBV1.2 HBV competent plasmid was kindly provided by Prof. Peijer Chen from National Taiwan University, and was constructed as previously described (Wu et al., 2017).

Commercial enzyme-linked immunosorbent assay kits for detecting HBsAg, HBeAg, and alanine aminotransferase (ALT), aspartate aminotransferase (AST) were acquired from Kehua Bio-Engineering and Beijing Solarbio Science & Technology, respectively. The PrimeScript™ RT Reagent Kit and TB Premix Ex Taq (Tli RNaseH Plus) were from Takara. The Cell Counting kit-8, Dual-Luciferase Reporter Assay Kit, and RNA-easy Isolation Reagent were from Vazyme Biotech. The QIAamp DNA Blood Mini Kit was from Qiagen.

Antibodies used for immunoblotting and immunofluorescence were as follows: anti-β-actin (#4967), anti-FOXO1(C29H4, #2880), anti-phosphorylated-FOXO1 (p-FOXO1, Ser256, #9461), anti-ERK (137F5, #4695), and anti-HNF4α (C11F12, #3113) from Cell Signaling Technology; anti-phosphorylated-ERK (p-ERK, T202/Y204, SAB5701896) from Sigma; anti-HBcAg (C1, EPR28251-34) from Abcam; anti-HBsAg (1023, sc-53299), anti-Pol (2C8, sc-81590), and anti-PGC-1α (D-5, sc-518025) from Santa Cruz Biotechnology.

### 2.2 Cytotoxicity assay

Cell viability was assessed using the Cell Counting kit-8 assay. Briefly, cells were seeded in 96-well plates at a density of 5×10^4^ cells per well. After 24 h of adherence, cells were treated with KP at serial concentrations (6.25 to 400 μM) or an equivalent volume of vehicle dimethyl sulfoxide for 48 h. Subsequently, 10 μL of Cell Counting kit-8 regent was added to each well and incubated for 30 min at 37°C. The absorbance at 450 nm was measured using a Bio-Rad microplate reader. Cell viability was expressed as a percentage relative to the vehicle-treated control group.

### 2.3 Analysis of HBV DNA levels

Intracellular HBV DNA: Cells cultured in 6-well plates were lysed with the ice-cold lysis buffer. The lysate was treated with 10% SDS and proteinase K (0.5 mg/mL) at 56 °C for 2 h. Total DNA was then extracted *via* phenol/chloroform (1:1) extraction, precipitated with ethanol using glycogen as a carrier, and resuspended in nuclease-free water.

Extracellular HBV DNA: Viral DNA was extracted from 200μL of the cell culture supernatant using the QIAamp DNA Blood Mini Kit according to the manufacturer’s instructions.

The purified DNA was subjected to quantitative polymerase chain reaction using HBV-specific primers (**Supplementary Table 1**) and TB Green Premix Ex Taq (Tli RNaseH Plus) on a QuantStudio real-time PCR system. HBV DNA levels were quantified against a standard curve generated from known amounts of a plasmid containing the full-length HBV genome.

### 2.4 Quantitative reverse transcription PCR

Total RNA was isolated from cells using the RNA-easy Isolation Reagent and reverse-transcribed into cDNA using the PrimeScript™ RT Reagent kit according to the manufacturer’s instructions. Quantitative polymerase chain reaction was performed with gene-specific primers listed in **Supplementary Table 1** and TB Green Premix Ex Taq (Tli RNaseH Plus). The relative expression levels of target genes were calculated using the comparative threshold cycle (2^-△△Ct^) method, with GAPDH serving as the endogenous control.

### 2.5 Enzyme-linked immunosorbent assay

The levels of HBsAg, HBeAg and ALT, AST secreted into the cell culture supernatant or from mouse serum were quantified using commercial enzyme-linked immunosorbent assay kits according to the manufacturer’s instructions. Absorbance was measured at 450 nm for HBsAg, HBeAg, and 505 nm for ALT, AST, respectively. The concentrations of ALT and AST were determined based on a standard curve.

### 2.6 Immunofluorescence analysis

HepG2.2.15 were seeded on coverslips in 6-well plates at a density of 2×10^5^ cells per well. After treatment, cells were fixed with 4% paraformaldehyde, permeabilized with 0.1% Triton X-100, and blocked with 5% bovine serum albumin. Cells were then incubated overnight at 4 °C with primary antibodies, followed by incubation with a fluorophore-conjugated secondary antibody for 1 h at room temperature. Nuclei were counterstained with DAPI. Images were acquired using a fluorescence microscope.

### 2.7 Immunohistochemistry analysis

Liver tissue samples from the right lobe of the mouse liver were fixed in 4% paraformaldehyde, embedded in paraffin, and sectioned. After deparaffinization, rehydration, and antigen retrieval, sections were blocked for endogenous peroxidase activity and incubated with primary antibodies overnight at 4 °C. This was followed by incubation with a HRP-conjugated secondary antibody. The signal was developed with a DAB substrate, and nuclei were counterstained with hematoxylin. Slides were visualized under a bright-field microscope.

### 2.8 Western blot analysis

Cells or homogenized liver tissues were lysed in RIPA buffer containing protease and phosphatase inhibitors. Equal amounts of protein were separated by SDS-PAGE and transferred onto PVDF membranes. After blocking, membranes were probed with specific primary antibodies overnight at 4 °C, followed by incubation with HRP-conjugated secondary antibodies. Protein bands were visualized using an enhanced chemiluminescence substrate on a Bio-Rad ChemiDoc imaging system and normalized to β-actin.

### 2.9 Dual-luciferase reporter assay

To assess HBV promoter activity, Huh7 cells were co-transfected in 24-well plates with a firefly luciferase reporter plasmid driven by the viral four promoters and a Renilla luciferase control plasmid. After 6 h, the medium was replaced with fresh medium containing KP or vehicle. 48 h post-transfection, cells were lysed, and luciferase activities were measured sequentially using the Dual-luciferase Reporter Assay Kit. Firefly luciferase activity was normalized to Renilla luciferase activity for each sample.

### 2.10 Animal experiments

All animal procedures were approved by the Ethics Committee of China Pharmaceutical University (Approval NO. IACUC-2023-09-011) and conducted in accordance with institutional guidelines.

Male C57BL/6 mice (5–6 weeks old, 18–20 g) from the Comparative Medicine Center of Yangzhou University were housed under standard conditions. An HBV persistence infection model was established *via* hydrodynamic injection of 10 μg the pAAV-HBV1.2 plasmid in a volume equivalent to 10% of the mouse body weigh into the tail vein within 5–8 s. One day post-injection, mice were randomly assigned to six groups (n=6 per group): (1) Model group (vehicle:0.5% sodium carboxymethyl cellulose); (2) KP low-dose (10 mg/kg), (3) KP medium-dose (20 mg/kg); (4) KP high-dose (40 mg/kg); (5) Combination (KP 40 mg/kg and ETV 1 mg/kg); (6) Positive control (ETV, 1 mg/kg). KP was suspended in 0.5% sodium carboxymethyl cellulose solution. Administrations were performed *via* oral gavage every other day for a total of nine times. Mice in the model and combination groups received corresponding volumes of vehicles.

### 2.11 Statistical analysis

All data are presented as the mean ± standard error of the mean (SEM) from at least three independent experiments. Statistical analyses were performed using GraphPad Prism 8.0 software. Comparisons among multiple groups were analyzed by one-way analysis of variance followed by an appropriate *post-hoc* test. A *p*-value of less than 0.05 (*p* < 0.05) was considered statistically significant.

## 3 Results

### 3.1 KP inhibits the HBV life cycle in Huh7D^hNTCP^ cells

The molecular structure of KP was displayed in **Figure 1**, and we first evaluated the potential cytotoxicity of KP in relevant hepatoma cell lines. Treatment with KP at various concentrations for 48 h resulted in half-cytotoxic concentration (CC_50_) values of 394.5 μM for Huh7D^hNTCP^, 354.0 μM for HepG2.2.15, and 380.9 μM for Huh7 cells (**Supplementary Figure 1**), indicating low cytotoxicity. Based on these results, non-cytotoxic concentrations of 1, 5, and 25 μM KP were selected for all subsequent *in vitro* experiments.

**Figure 2:**
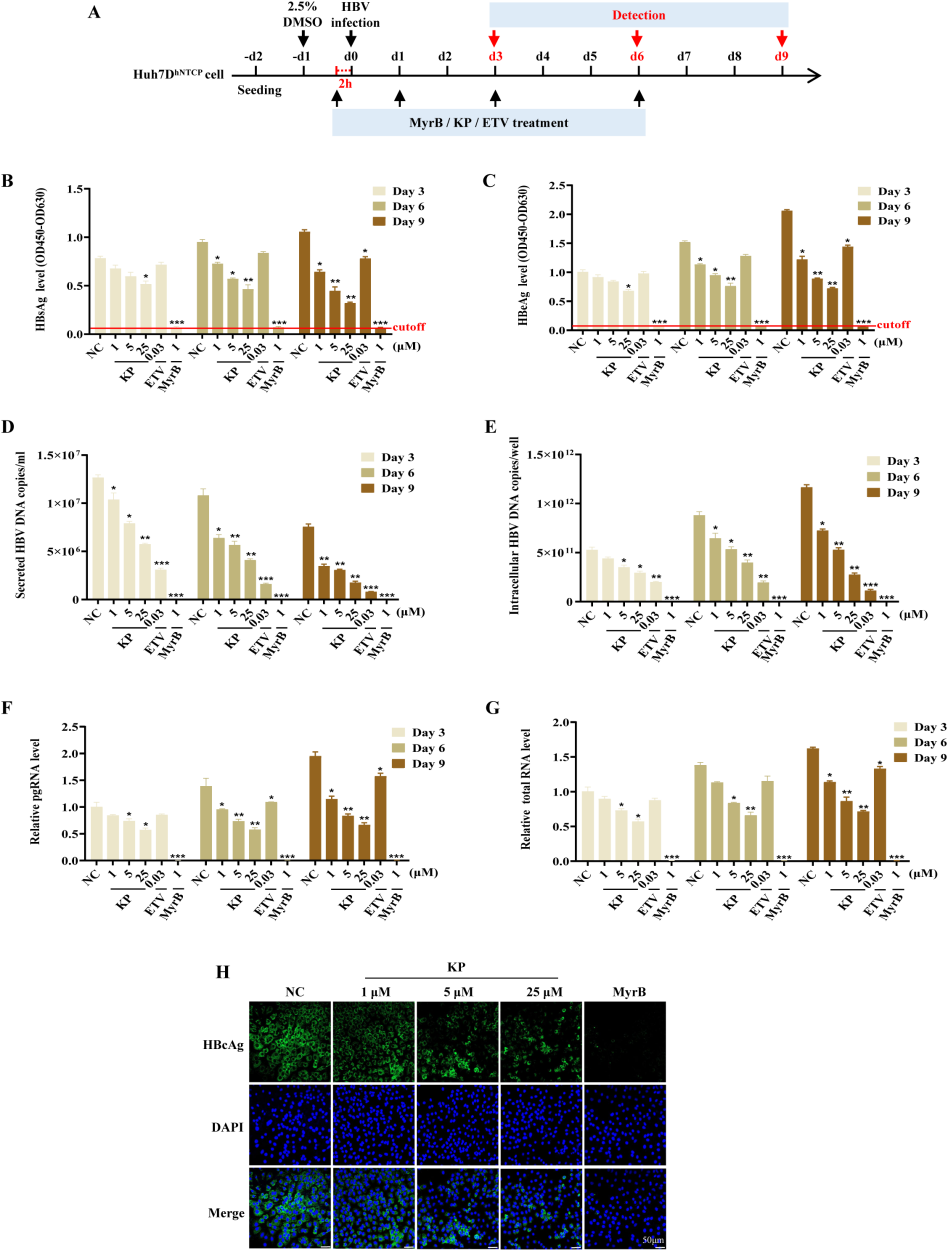
KP inhibits HBV replication in Huh7D^hNTCP^ cell. **(A)** Experimental timeline for KP treatment in HBV-infected Huh7D^hNTCP^ cells. Cells were infected with 1000 genome equivalents/cell of HBV in the presence of 8% polyethylene glycol 8000 and 2% dimethyl sulfoxide. **(B–G)** KP treatment reduces the levels of secreted HBsAg **(B)**, HBeAg **(C)**, and extracellular HBV DNA **(D)**, as well as intracellular HBV DNA **(E)**, pgRNA **(F)**, and total HBV RNA **(G)** in a dose- and time-dependent manner. Data were collected on days 3, 6, and 9 post-infection. **(H)** Immunofluorescence images showing the dose-dependent suppression of intracellular HBcAg (green) by KP. Nuclei were counterstained with DAPI (blue). *P < 0.05, **P < 0.01, ***P < 0.001.

To assess the anti-HBV activity of KP, Huh7D^hNTCP^ cells were infected with HBV for 24 h and then treated with KP according to the schedule outlined in **Figure 2A**. Myrcludex B (MyrB), an NTCP inhibitor, served as a negative control for HBV infection. KP treatment led to a potent, dose- and time-dependent reduction in the secretion of viral HBsAg, HBeAg, and extracellular HBV DNA into the supernatant (**Figures 2B–D**). Intracellular viral replication intermediates were similarly suppressed, with levels of intracellular HBV DNA, pgRNA, and total viral RNA significantly decreased in a dose- and time-dependent manner (**Figures 2E–G**). Additionally, Immunofluorescence analysis further confirmed that KP dose-dependently suppressed the expression of intracellular HBcAg in Huh7D^hNTCP^ cells (**Figure 2H**). While, the level of cccDNA was not affected by KP (**Supplementary Figure 2**).

### 3.2 KP suppresses HBV replication in HepG2.2.15 cells

**Figure 3:**
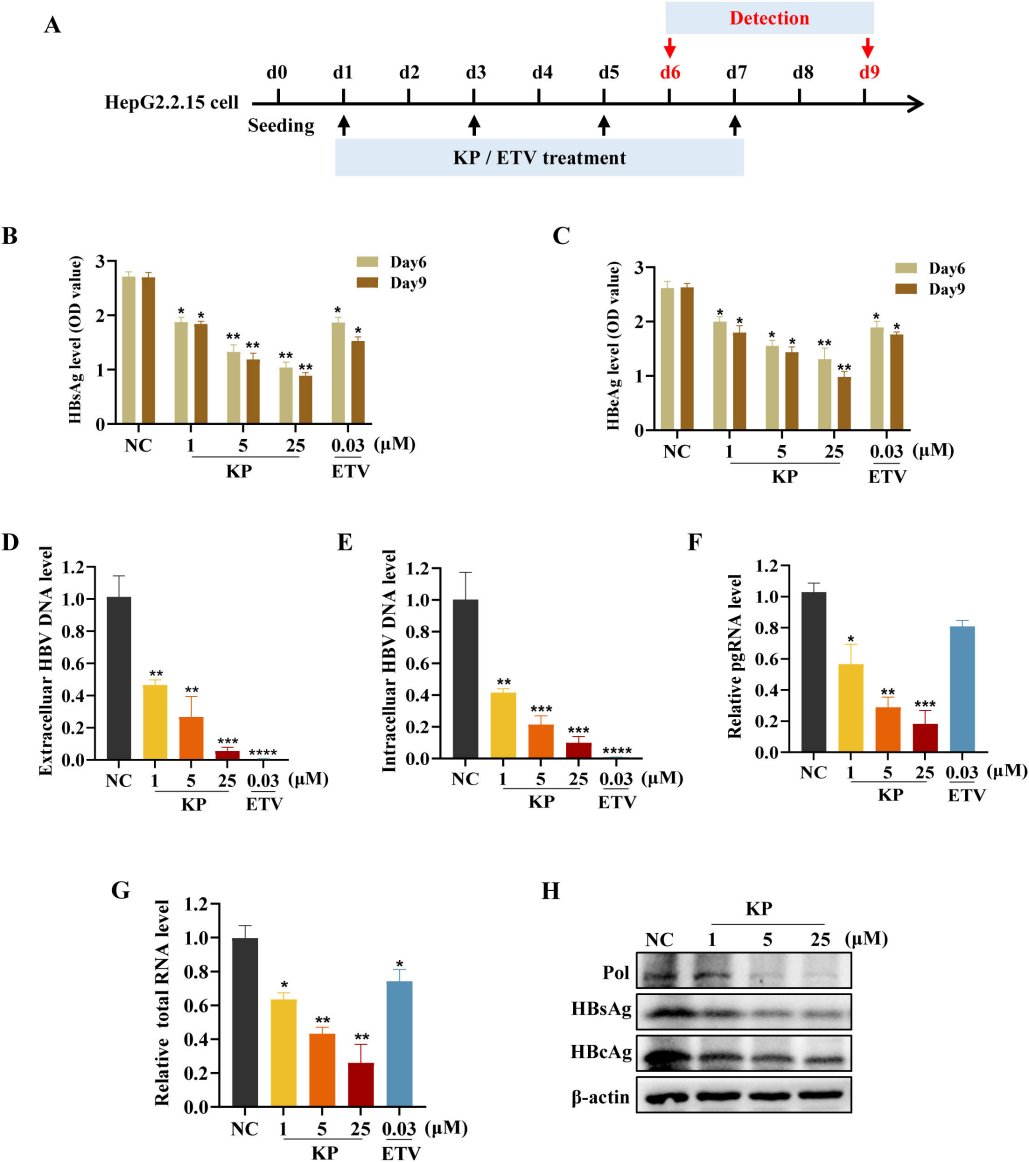
KP suppresses HBV replication in HepG2.2.15 cells. **(A)** Experimental timeline for KP treatment in HepG2.2.15 cells. **(B, C)** KP dose- and time-dependently inhibits the secretion of HBsAg **(B)** and HBeAg **(C)**. **(D, E)** KP reduces both extracellular **(D)** and intracellular **(E)** HBV DNA levels on day 9. **(F, G)** KP downregulates viral pgRNA **(F)** and total HBV RNA **(G)** levels on day 6. **(H)** Western blot analysis shows the dose-dependent reduction of viral polymerase (Pol), HBsAg, and HBcAg protein levels following KP treatment. *P < 0.05, **P < 0.01, ***P < 0.001, ****P < 0.0001.

The anti-HBV effect of KP was further validated in HepG2.2.15 cells, which stably support HBV replication. Cells were treated with KP every 48 h, and samples were collected at the indicated time points (**Figure 3A**). Consistent with the infection model, KP dose- and time-dependently inhibited the secretion of HBsAg and HBeAg in HepG2.2.15 cells (**Figures 3B, C**). Both extracellular and intracellular HBV DNA levels, as well as viral pgRNA and total RNA levels, were markedly downregulated by KP in a dose-dependent manner (**Figures 3D–G**). Notably, unlike the Pol inhibitor ETV, which primarily targets DNA synthesis, KP significantly reduced pgRNA levels (**Figure 3F**), suggesting an additional mechanism affecting viral transcription. Western blot analysis confirmed that KP dose-dependently reduced the expression of intracellular viral proteins, including Pol, HBsAg, and HBcAg in HepG2.2.15 cells (**Figure 3H**).

We next assessed the combined effect of KP and ETV. Co-treatment with 5 μM KP and 10 nM ETV reduced intracellular and extracellular HBV DNA levels by 87% and 84.73%, respectively. This inhibition was more pronounced than that achieved with 10 nM ETV alone (77% and 78.20% inhibition, respectively) (**Supplementary Figure 3**), suggesting a combined anti-HBV effect with ETV.

### 3.3 KP exhibits antiviral efficacy in a mouse model of persistent HBV replication

**Figure 4:**
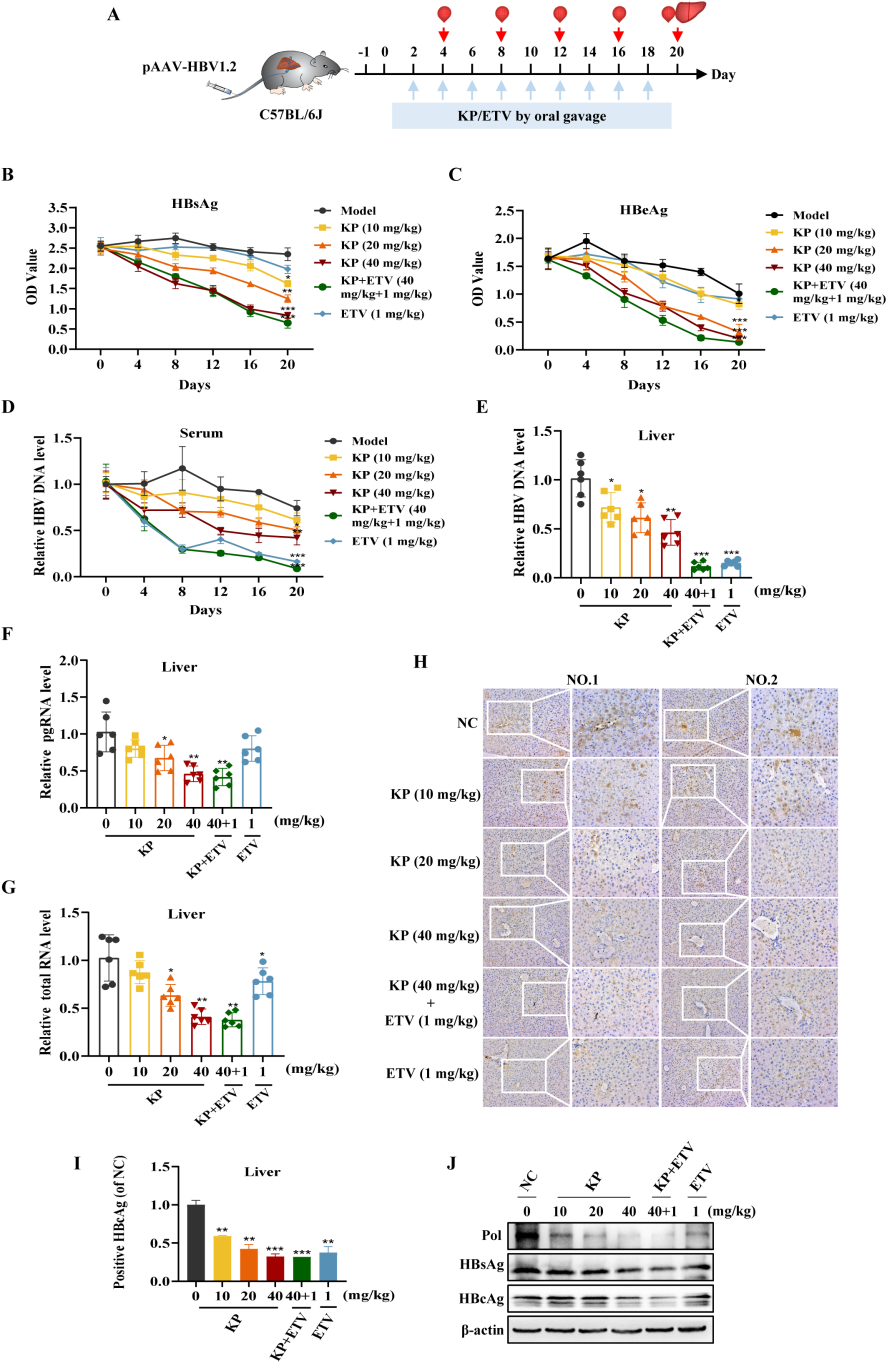
KP inhibits HBV replication in a mouse model of persistent infection. **(A)** Schematic of the hydrodynamic injection-based mouse model and treatment regimen. **(B-D)** Serum levels of HBsAg **(B)**, HBeAg **(C)**, and HBV DNA **(D)** are decreased by KP in a dose- and time-dependent manner. **(E–G)** Hepatic levels of HBV DNA **(E)**, pgRNA **(F)**, and total RNA **(G)** are similarly reduced. **(H)** Representative immunohistochemical staining of HBcAg in liver tissues. **(I)** Quantitative analysis of HBcAg-positive cells. **(J)** Western blot analysis of viral Pol, HBsAg, HBcAg protein levels in liver tissues. *P < 0.05, **P < 0.01, ***P < 0.001.

After established the *in vitro* activity of KP, we evaluated its efficacy *in vivo* using a hydrodynamic injection-based mouse model of chronic HBV infection. The experiment timeline is shown in **Figure 4A**. Serum levels of AST and ALT in model mice increased slightly in the early stage of infection, while remained within normal ranges, confirming that the hydrodynamic injection did not cause significant liver injury, and KP treatment did not induce hepatotoxicity (**Supplementary Figure 4**). Body weights were comparable across all group throughout the study (**Supplementary Figure 5**).

High serum HBsAg levels on day 0 confirmed successful model establishment (**Figure 4B**). KP administration induced a time- and dose-dependent decline in serum levels of HBsAg, HBeAg, and HBV DNA (**Figures 4B–D**). Consistent with the serum data, analysis of liver tissues revealed that KP dose-dependently reduced hepatic levels of HBV DNA, pgRNA, total viral RNA, and the viral proteins, including HBcAg, Pol, and HBsAg (**Figures 4E–J**). Notably, KP was more effective than ETV monotherapy in reducing viral pgRNA levels (**Figure 4F**). Furthermore, KP treatment dose-dependently attenuated the HBV-induced upregulation of hepatic pro-inflammatory cytokines TNF-α, IL-6, and IL-1β, with levels in the high-dose KP group even falling below those of the normal control (**Supplementary Figure 6**), indicating combined antiviral and anti-inflammatory properties of KP.

### 3.4 KP inhibits HBV Cp activity *via* the ERK-FOXO1 axis

**Figure 5:**
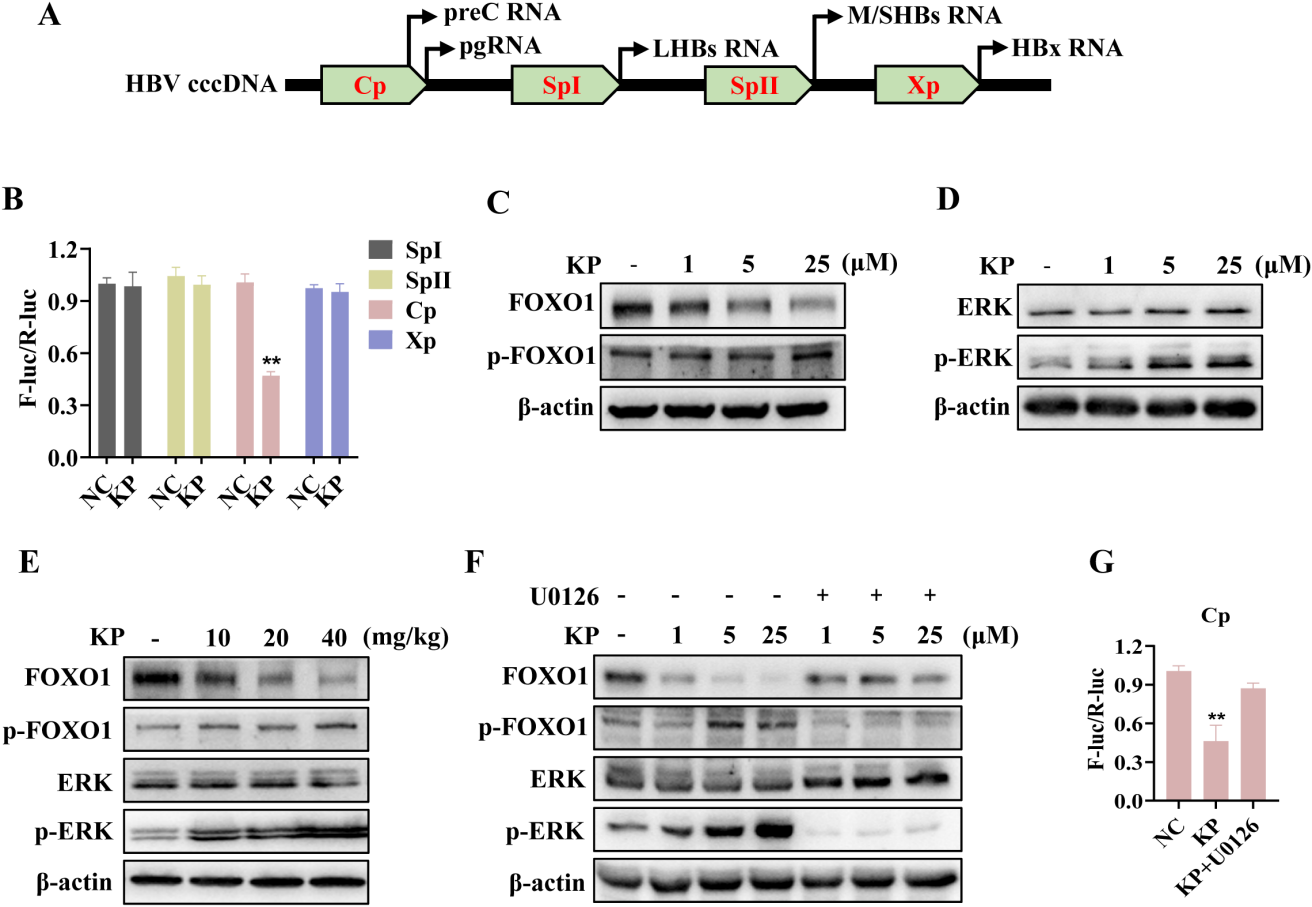
KP inhibits the HBV core promoter (Cp) activity *via* the ERK/FOXO1 axis. **(A)** Schematic diagram of the HBV promoter regions. **(B)** Dual-luciferase reporter assays show that KP (25 μM) specifically suppresses the activity of the Cp, with minimal effects on SpI, SpII, or Xp in Huh7 cells. **(C, D)** Western blot analysis reveals that KP treatment in HepG2.2.15 cells increases phosphorylation of FOXO1 (p-FOXO1) **(C)** and ERK (p-ERK) **(D)**, while decreases FOXO1 **(C)** in a dose-dependent manner. **(E)** Consistent effects are observed in mouse liver tissues, with KP upregulating p-ERK and p-FOXO1, while downregulating FOXO1. **(F)** The ERK inhibitor U0126 blocks KP-induced phosphorylation of ERK and FOXO1 in HepG2.2.15 cells. **(G)** U0126 (5 μM) reverses the KP-mediated suppression of Cp activity in Huh7 cells. **P < 0.01.

The observed suppression of viral RNA, combination with ETV, and reduction in viral proteins suggested that KP may target HBV transcription. HBV transcription is governed by four viral promoters: the Cp, two surface promoters (SpI, SpII), and the X promoter (Xp), which regulates the transcription of viral X protein (HBx) (**Figure 5A**). Dual-luciferase reporter assays demonstrated that KP predominantly inhibited the activity of the Cp, with minimal effects on the other promoters (**Figure 5B**).

As the viral Cp is regulated by host factors, we examined key transcriptional regulators, including PGC-1α, HNF4α, FOXO1 (Shlomai and Shaul, 2009a; Zheng et al., 2004; Curtil et al., 2014). In HepG2.2.15 cells, KP treatment dose-dependently decreased FOXO1 protein levels while increasing its phosphorylated form (p-FOXO1) (**Figure 5C**). In contrast, the protein levels of PGC-1α and HNF4α were unchanged (**Supplementary Figure 7**). FOXO1 is a nuclear transcription factor known to enhance HBV transcription, and its phosphorylation induces cytoplasmic translocation and functional inactivation (Butt et al., 2011; Xing et al., 2018; Shlomai and Shaul, 2009b). Since ERK is a known upstream kinase regulating FOXO1 phosphorylation in hepatocytes (Jiao et al., 2013), we examined p-ERK as its activation status. KP treatment dose-dependently increased p-ERK levels (**Figure 5D**). Consistent with the *in vitro* findings, KP upregulated p-ERK and p-FOXO1 while downregulated FOXO1 in mouse liver tissues (**Figure 5E**). To directly link this pathway to the antiviral effect, we used the specific ERK inhibitor U0126. Pre-treatment with U0126 abolished KP-induced increases in p-ERK and p-FOXO1, and prevented the decrease in FOXO1 (**Figure 5F**). Crucially, U0126 also reversed the KP-mediated suppression of viral Cp activity by the luciferase assay (**Figure 5G**).

### 3.5 The ERK inhibitor U0126 rescues KP-mediated suppression of HBV replication

**Figure 6:**
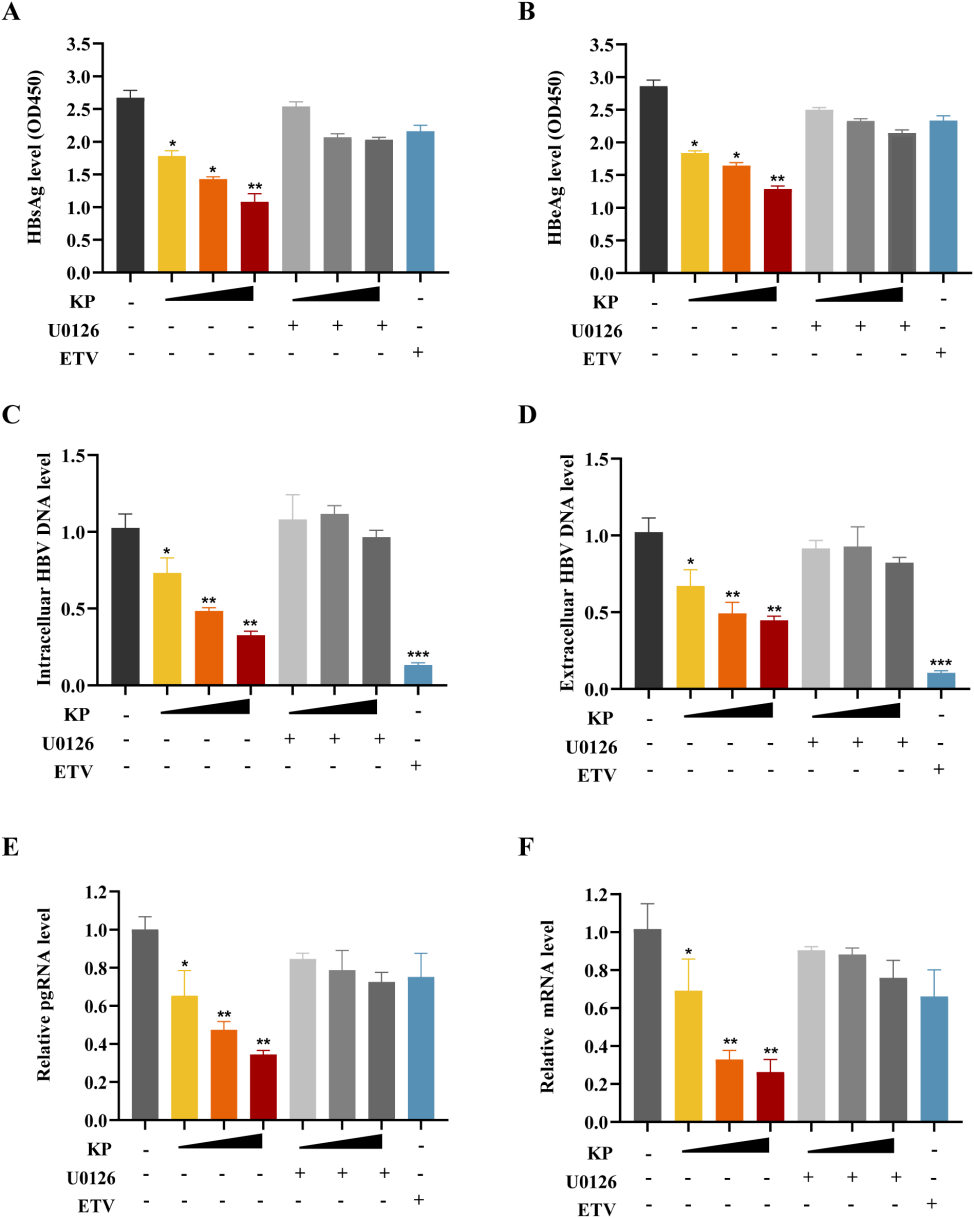
The ERK inhibitor U0126 rescues the anti-HBV effects of KP. HepG2.2.15 cells were treated with KP (0, 1, 5, 25 μM), the ERK inhibitor U0126 (5 μM), the polymerase inhibitor ETV (0.03 nM), or their combinations. **(A, B)** Levels of secreted HBsAg **(A)** and HBeAg **(B)**. **(C, D)** Levels of intracellular **(C)** and extracellular **(D)** HBV DNA. **(E, F)** Levels of viral pgRNA **(E)** and total HBV RNA **(F)**. U0126 co-treatment significantly attenuates the suppression of all viral markers by KP, confirming the dependency of KP’s action on ERK signaling. *P < 0.05, **P < 0.01, ***P < 0.001.

A final rescue experiment was performed to confirm the functional role of the ERK/FOXO1 pathway in the anti-HBV activity of KP. We assessed viral replication markers after co-treatment with KP and ERK inhibitor U0126. Although KP dose-dependently inhibited the levels of viral HBsAg, HBeAg, HBV RNA, and DNA, co-treatment with U0126 significantly attenuated this suppression across all viral markers (**Figure 6**). This reversal confirmed that the anti-HBV effect of KP was primarily mediated through activation of the ERK/FOXO1 signaling pathway.

## 4 Discussion

The primary endpoint of clinical therapy for chronic HBV infection is a functional cure. Current treatments, including existing clinical regimens and emerging agents such as oligonucleotides, siRNA, and capsid assembly modulators, have not yet achieved substantial functional cure rates (Shechter et al., 2025). Critically, these approaches rarely eliminate the highly stable cccDNA reservoir in hepatocytes, which is essential for a complete cure (Xia and Guo, 2020). Consequently, novel strategies targeting cccDNA transcription are urgently needed. Natural products, with their broad biological activities and favorable safety profiles, represent a promising source of such strategies. They exert anti-HBV effects through multiple pathways, including direct interference with the viral life cycle and modulation of host factors (Deng et al., 2023; Pan et al., 2023). Some compounds, such as alpha-glucosidase inhibitors (Hayakawa et al., 2020) and dicoumarol (Cheng et al., 2021), can silence cccDNA by regulating its transcriptional activity. In line with this direction, our study demonstrates that the dietary flavonol KP inhibits the activity of the HBV Cp, thereby suppressing viral transcription from cccDNA and subsequent replication.

The transcriptional activity of HBV Cp is regulated by various host factors (Yang et al., 2020; Ibrahim et al., 2021; Wei et al., 2022). FOXO1, a key mediator of hepatic glucose metabolism, has been showed to bind and activate the HBV Cp in the nucleus (Shlomai and Shaul, 2009b). Phosphorylation of FOXO1 triggers its translocation to the cytoplasm and consequent loses of transcriptional activity (Xing et al., 2018; Butt et al., 2011), a process that can be induced by ERK phosphorylation (Jiao et al., 2013). Since KP is known to regulate p-ERK levels in various cell models (Chien et al., 2019; Li et al., 2021; Wu et al., 2024), we hypothesized that it might similarly regulate FOXO1 *via* ERK in hepatocytes. Our results confirmed that KP activates the ERK/FOXO1 pathway, leading to transcriptional repression of the viral Cp. This study thus establishes a novel link between KP’s bioactivity and HBV replication. Mechanistically, pharmacological targeting of the ERK/FOXO1 axis could represent a promising dual strategy to suppress viral replication and promote a repressive state on the viral cccDNA minichromosome, which is considered as a more effective strategy to achieve a functional cure for HBV. Besides the transcriptional repression of viral cccDNA, post-translational modifications like ubiquitination are also involved in HBV life cycle and virus-related liver disease progression (Kar et al., 2024). Therefore, convergence of diverse host-targeting may offer a multifaceted approach to achieve a more durable control of chronic HBV infection, and future investigations could explore whether KP-induced ERK/FOXO1 activation subsequently influences the host factors or whether combining KP with agents that modulate specific post-translational modifications would yield synergistic effects on viral replication.

Plant-derived compounds such as KP are increasingly recognized for their therapeutic potential in liver diseases, demonstrating hepatoprotective effects through diverse molecular mechanisms in preclinical models (Nizinski et al., 2025; Alkandahri et al., 2023). In this study, KP not only exhibited potent anti-HBV activity but also showed low cytotoxicity in hepatocytes *in vitro* and *in vivo*, supporting its promise as a candidate for antiviral development. However, longer-term preclinical and clinical studies are necessary to fully evaluate its efficacy and safety profile, such as validation in primary human hepatocytes. Beyond its direct suppression of Cp activity, it remains to be explored whether KP indirectly regulates the HBV life cycle *via* host cell pathways, such as inducing complete autophagic flux. Future investigations should also explore strategies to enhance its targeted delivery and bioavailability, such as the development of nanoformulations (Chandekar et al., 2022), to fully realize KP’s anti-HBV potential.

In summary, this study demonstrates that KP exerts potent anti-HBV activity *in vitro* and *in vivo* and acts in combination with the nucleostide analog ETV. Mechanistically, KP promotes ERK-dependent phosphorylation of FOXO1, leading to suppression of viral Cp activity and cccDNA transcription. The crucial role of this pathway was further confirmed by the rescue of viral replication upon treatment with the ERK inhibitor U0126.

## Data Availability

The original contributions presented in the study are included in the article/**Supplementary Material**. Further inquiries can be directed to the corresponding authors.
