# Supplementary material for: Kaempferol inhibits hepatitis B virus replication via ERK/FOXO1 pathway-mediated suppression of the viral core promoter
Source: Front Cell Infect Microbiol. 2026 Feb 19;16:1780484. doi: 10.3389/fcimb.2026.1780484 (PMC12960505; doi:10.3389/fcimb.2026.1780484)
Supplement: Supplementary file 1 [file Table1.docx]

Supplementary Material

# 1 Supplementary Figures and Tables

## 1.1 Supplementary Tables

# Supplementary Table 1. Sequences of the primers

| **Primer** | **Sequences** |
| --- | --- |
| Total HBV RNA-F | 5’-TCACCAGCACCATGCAAC-3’ |
| Total HBV RNA-R | 5’-AAGCCACCCAAGGCACAG-3’ |
| HBV pgRNA-F | 5’-CTGGGTGGGTGTTAATTTGG-3’ |
| HBV pgRNA-R | 5’-TAAGCTGGAGGAGTGCGAAT-3’ |
| HBV DNA-F | 5’-ACCAATCGCCAGTCAGGAAG-3’ |
| HBV DNA-R | 5’-ACCAGCAGGGAAATACAGGC-3’ |
| HBV cccDNA-F | 5'-CTTCTCATCTGCCGGACC-3' |
| HBV cccDNA-R | 5'-CACAGCTTGGAGGCTTGA-3' |
| hGAPDH-F | 5’-AAATCAAGTGGGGCGATGCTG-3’ |
| hGAPDH-R | 5’-GCAGAGATGATGACCCTTTTG-3’ |
| mTNF-α-F | 5’-ACAAGGCTGCCCCGACTAC-3’ |
| mTNF-α-R | 5’-TGGGCTCATACCAGGGTTTG-3’ |
| mIL-6-F | 5’-ACCACTCCCAACAGACCTGTCT-3’ |
| mIL-6-R | 5’-CAGATTGTTTTCTGCAAGTGCAT-3’ |
| mIL-1β-F | 5’-CTTTCCCGTGGACCTTCCA-3’ |
| mIL-1β-R | 5’-CTCGGAGCCTGTAGTGCAGTT-3’ |
| mGAPDH-F | 5’-GAGCTGAACGGGAAGCTCAC-3’ |
| mGAPDH-R | 5’-AGTGTAGCCCAAGATGCCCT-3’ |

## 1.2 Supplementary Figures


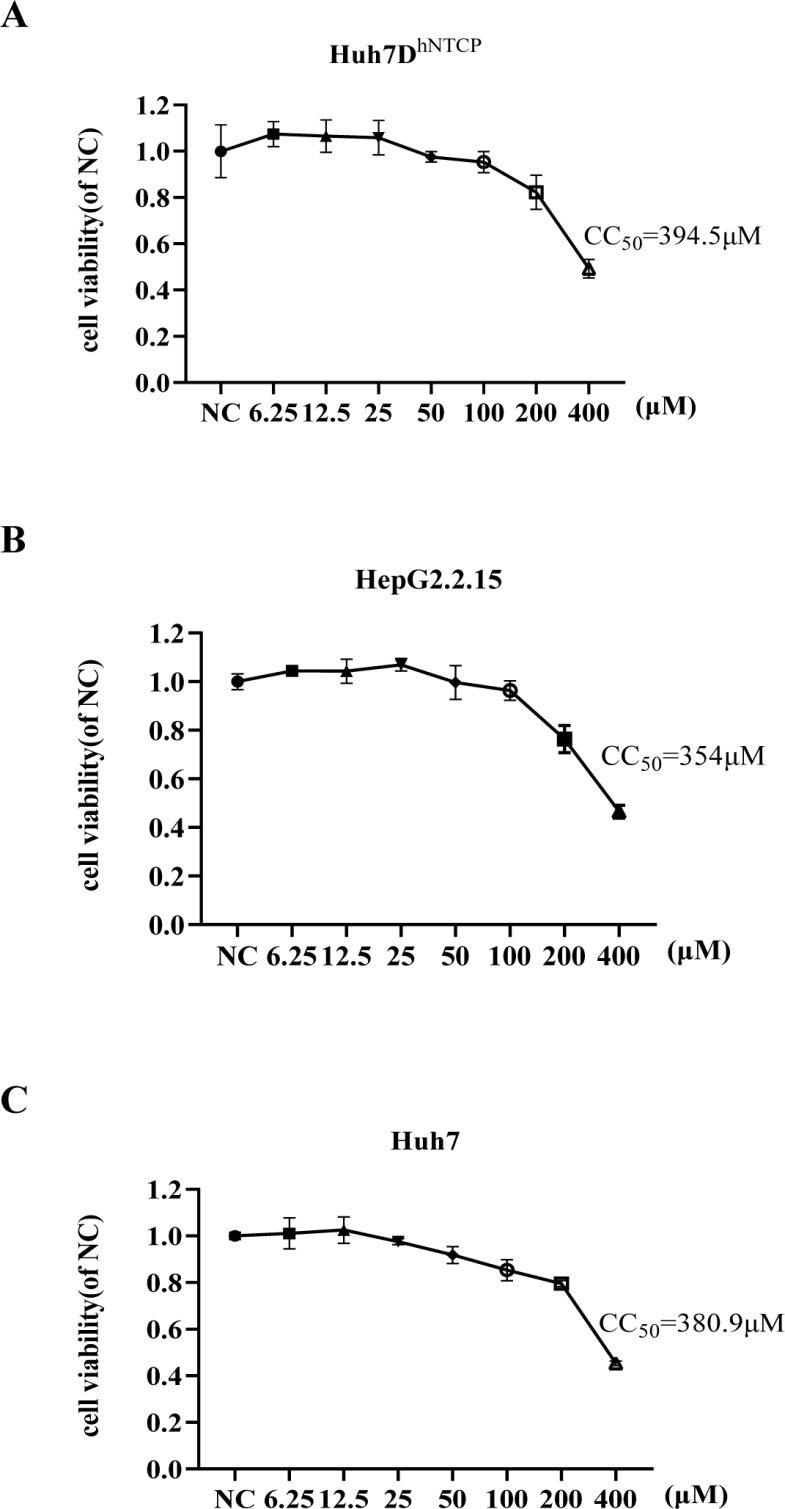


**Supplementary Figure 1. Cytotoxicity of kaempferol (KP) in hepatoma cell lines. Cell viability of **(A)** Huh7D^hNTCP^, **(B)** HepG2.2.15, and **(C)** Huh7 cells treated with the indicated concentrations of KP was assessed by** Cell Counting kit-8 assay. The half-cytotoxic concentration (CC_50_) values were 394.5 μM for Huh7D^hNTCP^, 354.0 μM for HepG2.2.15, and 380.9 μM for Huh7 cells.

**
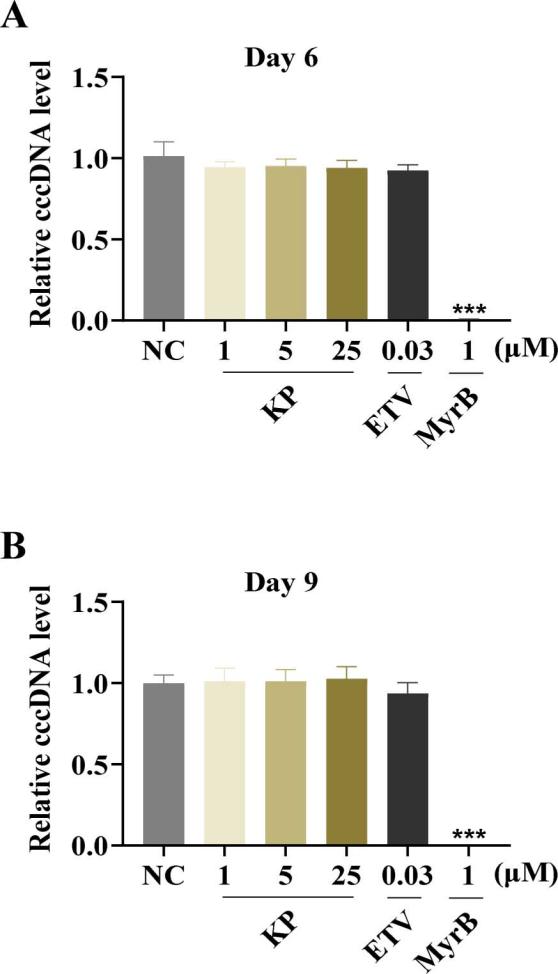
**

**Supplementary Figure 2.** HBV cccDNA levels in Huh7D^hNTCP^ cells. Cells were collected to examine the level of HBV cccDNA at day 6 **(**A**) and** 9 **(**B**)** after HBV infection by qPCR assay. Using gapdh as the loading control and the relative level was compared to NC.

****
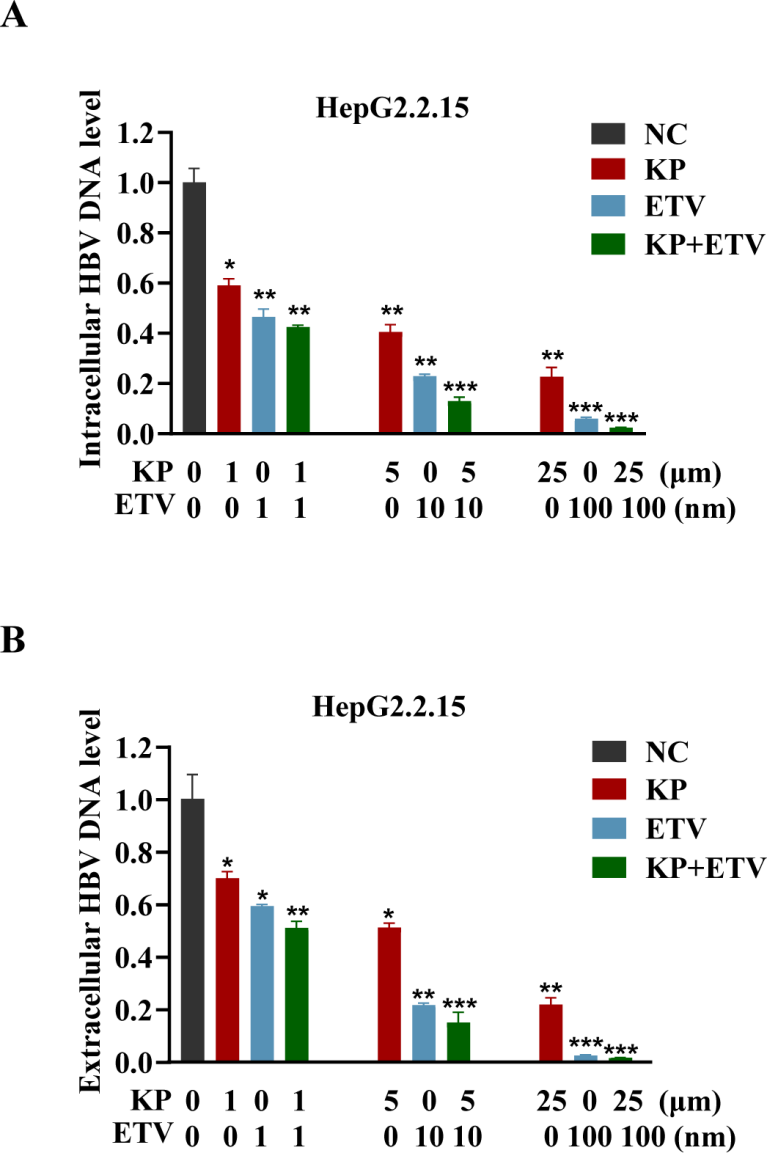
****

**Supplementary Figure **3.**** KP enhances the inhibitory effect of entecavir (ETV) on HBV DNA. **(A)** Intracellular and **(B)** extracellular HBV DNA levels in HepG2.2.15 cell treated with KP, entecavir (ETV), or their combination, as measured by quantitative polymerase chain reaction and compared to the negative control. * *P* < 0.05, ** *P* < 0.01, *** *P* < 0.001.


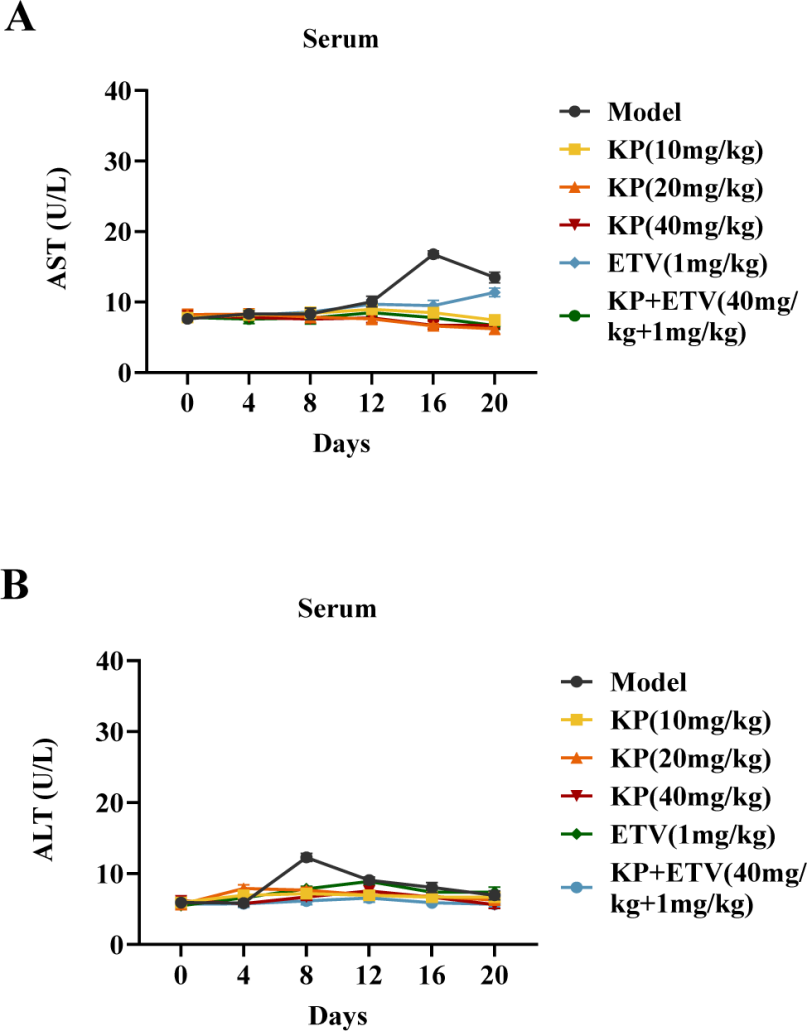


**Supplementary Figure **4.** Serum** levels of liver function markers in mice. Serum **(A)** aspartate aminotransferase (AST) and **(B)** alanine aminotransferase (ALT) were measured using a commercial enzyme-linked immunosorbent assay kit.


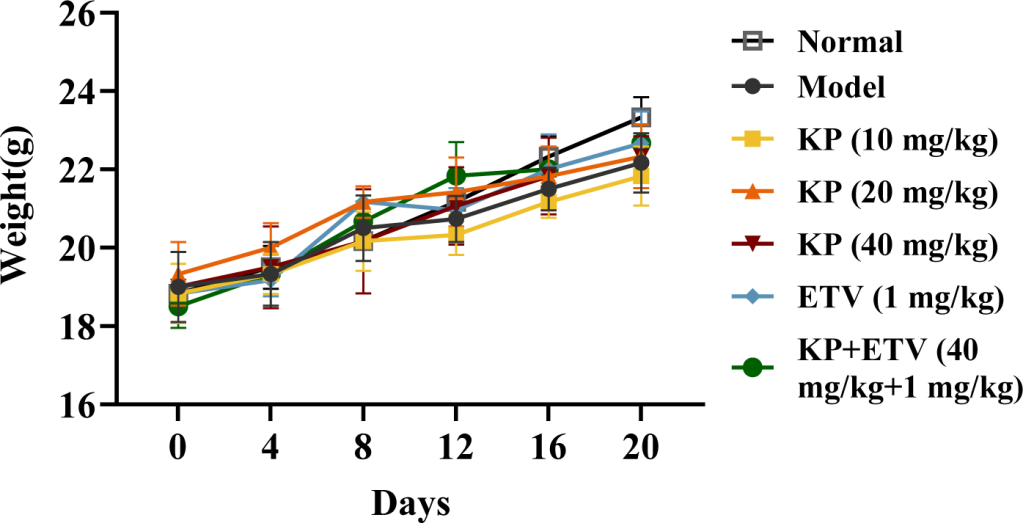


**Supplementary Figure **5.** Body weight changes in mice during the treatment period. Body weights were recorded at the** indicate time points for each treatment group.


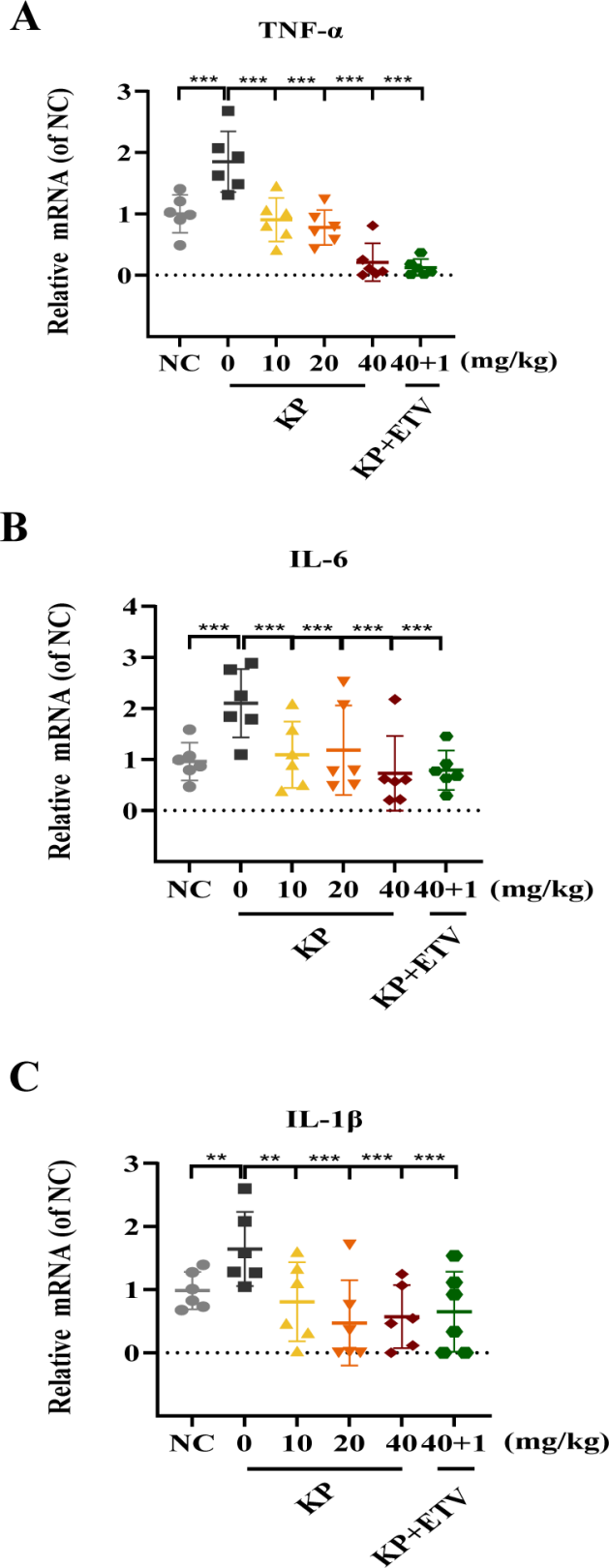


**Supplementary Figure **6.**** KP reduces the expressions of pro-inflammatory cytokines in the mouse liver tissues. After 20 days of consecutive administration with indicated concentrations of KP and ETV, the mRNA expression levels of **(A)** TNF-α, **(B)** IL-6, and **(C)**IL-1β in mouse liver tissues were determined by RT-qPCR. Using normal mice as negative control (NC). Data were analyzed between each group (n = 6) and 0 mg/kg KP group by one-way ANOVA; **P* < 0.01, ***P* < 0.001.

**
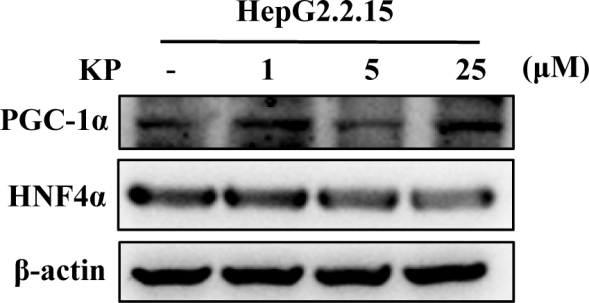
**

**Supplementary Figure **7.** Effects of KP on t**he protein levels of host transcription factors PGC-1α and HNF4α. HepG2.2.15 cells were treated with indicated concentrations of KP. Protein levels of PGC-1α and HNF4α were analyzed by western blotting. β-actin served as the loading control.
